# Clinical significance and biological role of L1 cell adhesion molecule in gastric cancer

**DOI:** 10.1038/s41416-019-0646-8

**Published:** 2019-11-22

**Authors:** Takashi Ichikawa, Yoshinaga Okugawa, Yuji Toiyama, Koji Tanaka, Chengzeng Yin, Takahito Kitajima, Satoru Kondo, Tadanobu Shimura, Masaki Ohi, Toshimitsu Araki, Masato Kusunoki

**Affiliations:** Department of Gastrointestinal and Pediatric Surgery, Mie University Graduate School of Medicine, Tsu, Japan

**Keywords:** Prognostic markers, Surgical oncology

## Abstract

**Background:**

L1 cell adhesion molecule (L1CAM) is highly expressed in malignant tumours and might play a pivotal role in tumour progression.

**Methods:**

We analysed by immunohistochemistry L1CAM protein expression in formalin-fixed, paraffin-embedded specimens from 309 GC patients. We performed propensity score matching (PSM) analysis to clarify the prognostic impact of L1CAM in GC patients. We evaluated L1CAM gene expression in fresh frozen specimens from another group of 131 GC patients to establish its clinical relevance. The effects of changes in L1CAM were investigated in vitro and in vivo.

**Results:**

L1CAM was mainly expressed in tumour cells of GC tissues. Elevated L1CAM expression was an independent prognostic factor for overall and disease-free survival, and an independent risk factor for distant metastasis in GC patients. PSM analysis showed that high L1CAM expression was significantly associated with poor prognosis. L1CAM gene expression using fresh frozen specimens successfully validated all of these findings in an independent cohort. Inhibition of L1CAM suppressed cell proliferation, cycle progress, invasion, migration and anoikis resistance in GC cells. Furthermore, L1CAM inhibition suppressed the growth of peritoneal metastasis.

**Conclusion:**

L1CAM may serve as a feasible biomarker for identification of patients who have a high risk of recurrence of GC.

## Background

Gastric cancer (GC) is the fourth most common cancer worldwide, and the second leading cause of cancer death in men and the fourth in women.^1,2^ Although surgical techniques and adjuvant chemotherapy have substantially improved recently and rate of early detection by endoscopy has increased, the overall 5-year survival rate remains dismal.^1^ Therefore, biomarkers are needed to identify patients with high risk of recurrence and prognosis for providing benefits from closed follow-up and intensive treatment in GC patients.

L1 cell adhesion molecule (L1CAM) is a 200–220-kDa type I membrane glycoprotein of the immunoglobulin family and has an important function in development of the nervous system by regulating cell adhesion and migration.^3–6^ L1CAM is also important for adhesion and migration of tumour cells,^7–9^ and the oncogenic activity of L1CAM has been demonstrated experimentally in various malignancies.^10,11^

In this study, we systematically evaluated the prognostic impact and biomarker potential of L1CAM expression using various statistical methods and clinical specimens, including both FFPE and fresh frozen samples, and clarified the clinical burden of L1CAM expression in GC patients. We also investigated the biological features of L1CAM in GC using a series of in vitro and in vivo experiments.

## Methods

### Tissue samples and patient characteristics

Formalin-fixed and paraffin-embedded (FFPE) samples were obtained from 309 patients (218 men, 91 women, median age 68 years, age range 18–90 years) with primary GC for immunohistochemical measurement of L1CAM protein expression (FFPE cohort). Patients were enrolled between 2005 and 2011 at the Department of Gastrointestinal Surgery, Mie University Hospital, Japan. The patient age ranged from 18 to 90 years. Patients who underwent neoadjuvant therapy, were treated by endoscopic mucosal resection, and had non-gastric carcinomas were excluded. The patients included 218 men and 91 women with a median age of 68 years. Clinicopathological findings were based on tumour–node–metastasis (TNM) classification. There were 151 patients with stage I GC, 61 with stage II and 56 with stage III. Forty-one patients with distant metastases were classified as having stage IV GC. There were 197 patients with intestinal-type GC and 112 with diffuse type. Postoperative follow-up data were obtained from all patients, and the median follow-up duration was 39.6 months (range: 1–124 months).

On the other hand, 262 gastric specimens were preserved immediately after surgical resection in RNA later (Qiagen, Chatsworth, CA) and stored at −80 °C until RNA extraction for investigating L1CAM gene expression by real-time polymerase chain reaction (PCR) (fresh frozen cohort). These fresh frozen samples were obtained from Department of Gastrointestinal Surgery, Mie University Hospital, Japan. The specimens were from 131 patients (103 men, 28 women, median age 69 years) who were at the hospital between 2000 and 2005. Clinicopathological findings were based on the TNM classification. There were 18 patients with stage I, 19 with stage II and 57 with stage III GC. Thirty-seven patients with distant metastases were classified as having stage IV GC. There were 94 patients with intestinal-type GC and 37 with diffuse-type. Postoperative follow-up data were obtained from all patients, and the median follow-up duration was 26 months (range: 1–60.5 months).

All of enrolled patients in both cohorts were followed up after initial hospital discharge, with physical examination and tumour marker assays (carcinoembryonic antigen and carbohydrate antigen 19-9) performed every 1–3 months and computed tomography every 6 months. Endoscopic examinations were performed when necessary. None of the patients received preoperative treatment such as radiotherapy or chemotherapy. Written informed consent was obtained from all patients in accordance with guidelines approved by the Institutional Review Board of Mie University Hospital.

### Immunohistochemistry

FFPE sections (2–3 µm thickness) from 309 GC patients were used for immunohistochemical analysis of L1CAM expression. Following deparaffinisation and dehydration, specimens were boiled in 10 mM sodium citrate buffer to unmask antigens. Specimens were then blocked and incubated with primary antibody overnight at 4 °C. Antibody binding was detected by horseradish peroxidase Envision kit (Dako Cytomation, Glostrup, Denmark). All sections were counterstained with haematoxylin, as previously described.^12^ Primary antibody against L1CAM (Thermo Scientific, Waltham, MA) was diluted 1:50. Furthermore, to clarify the distribution of L1CAM expression in cancerous tissues, we performed immunohistochemical analysis using primary antibody against pan-cytokeratin (AE1/AE3; Abcam, Cambridge, UK) with dilution of 1:100. Positive and negative controls were also run simultaneously.

### Evaluation of immunohistochemistry

L1CAM expression in stained FFPE sections was analysed separately by two expert pathologists without knowledge of the clinicopathological or survival data of any of the patients. Expression of L1CAM was evaluated by scanning the entire tissue specimen under low-power magnification (×40) and confirmed under high-power magnification (×200 and ×400). As previously described,^13^ an immunoreactivity scoring system was applied using the following criteria: (A) 0, fraction of positive stained cells ≤ 5%; 1, 6%–25%; 2, 26%–50%; 3, 51%–75% and 4, >75%; (B) intensity of staining: 0, colourless; 1, pallide-flavens; 2, yellow; and 3, brown. Scores obtained from A and B were multiplied together to make the staining score according to the proportion and intensity of positively stained cancer cells. Specimens were rescored if the difference between the scores by the two pathologists was >3.

### Total RNA extraction and cDNA synthesis

The fresh frozen specimens were homogenised using a Mixer Mill MM 300 homogeniser (Qiagen, Chatsworth, CA). Total RNA from tissues and cell lines was isolated using an RNeasy mini kit (Qiagen). The concentration and quality of RNA were measured by UV absorbance at 260 and 280 nm, and OD_260/280_ ratios of 1.8–2.1 were considered to be adequate. cDNA was synthesised from 5.0 µg total RNA with a random hexamer and Superscript III Reverse Transcriptase (Invitrogen, Carlsbad, CA).

### Quantitative real-time reverse transcription-PCR

We performed quantitative real-time reverse transcription (RT)-PCR analysis using the StepOne™ Real Time PCR System (Applied Biosystems, Foster City, CA). Primers for L1CAM and GAPDH were designed by Primer 3 software (Biology Workbench version 3.2, San Diego Supercomputer Center, University of California, San Diego, CA), as previously described.^12^ The sequences used were: L1CAM: forward, CAGAGGTTCCAGGGCATCTA; reverse, CTGTCTCCTTTGGCCACTTG; GAPDH: forward, GGAAGGTGAAGGTCGGAGTC; reverse, AATGAAGGGGTCATTGATGG. PCR was performed with Power SYBR Green PCR Master Mix (×2) (Applied Biosystems). The following cycling conditions were used: 95 °C, 10 min, 40 cycles at 95 °C for 15 s and 60 °C for 1 min.

### Relative L1CAM levels

The relative gene expression levels for L1CAM were determined by the standard curve method, as previously described.^14^ Standard curves and linear equations were generated using 5-fold serial dilutions of random-primed qPCR Human Reference cDNA (Takara Bio, Clontec), which was prepared from a mixture of total RNAs collected from normal adult tissues. Within the range analysed, all standard curves were linear with an acceptable correlation coefficient (*R*^2^). The extent of target gene expression was calculated from the standard curve, and the cDNA in each sample was quantitatively normalised with respect to the GAPDH gene, which served as an internal control. Finally, the target gene mRNA levels were expressed as respective gene ratios relative to GAPDH mRNA levels. Real-time PCR assays were performed in duplicate for each sample, and the mean values were used to calculate gene expression levels.

### Cell lines

The human GC cell lines MKN7 (intestinal type), MKN74 (intestinal type), MKN45 (diffuse type), KATO III (diffuse type) and NUGC3 (diffuse type) were obtained from the Cell Recourse Center for Biomedical Research, Institute of Development, Aging and Cancer (Tohoku University, Sendai, Japan). The authenticity of these cell lines was routinely monitored by analysing a series of genetic and epigenetic markers specific for each cell line. These cell lines were maintained in RPMI-1640 medium supplemented with 10% foetal bovine serum and antibiotics at 37 °C in a 5% humidified CO_2_ atmosphere.

### Western blot analysis

Western blotting was performed as described previously.^15^ Immobilon membranes (Millipore, Billerica, MA) were incubated with the respective anti-human primary antibody at the recommended dilution [anti-L1CAM (Thermo Scientific), β-actin (Santa Cruz Biotechnology, Dallas, USA)].

### Immunofluorescence

Primary antibody for L1CAM (Abcam, 1:100, described above) were incubated overnight at 4 °C as first antibodies. After washing the sections five times for 5 min, Alexa Fluor* 546 goat anti-mouse IgG (1:200, Invitrogen, Renfrew, UK) as secondary antibodies were incubated for 1 h at room temperature. Nuclear staining was done with 4′,6′-diamidino-2-phenylindole dihydrochloride (DAPI) (ProLong Gold Antifade Reagent with DAPI; Invitrogen). Confocal images were acquired by IX71 inverted microscopy with a DP70 digital camera system (Olympus, Center Valley, PA, USA), as previously described.^16^

### L1CAM RNA interference studies

L1CAM-specific siRNA (Silencer^®^ Select Validated siRNA, standard purity) and negative control siRNA (Silencer™ Negative Control siRNA) were purchased from Ambion (Austin, TX). Transfections were performed by mixing cell suspensions with siRNA oligonucleotides (30 nM), Opti-MEM I (Invitrogen) and Lipofectamine RNAiMAX (Invitrogen) before cell plating, as previously described.^14^ Cells were maintained in a humidified atmosphere, and assays were performed after 24 h incubation.

### Cell proliferation assay and cell cycle analysis

Cell proliferation was evaluated using a WST-8 [2-(2-methoxy-4-nitrophenyl)-3-(4-nitrophenyl)-5-(2, 4-disulfophenyl)-2H-tetrazolium, monosodium salt] colorimetric assay. Further information was described in Supplementary file. For cell cycle analysis, the DNA content of L1CAM siRNA- and control siRNA-transfected GC cells was evaluated using the Muse^TM^ Cell cycle assay kit (Millipore, Billerica, MA) according to the manufacturer’s instructions using the Muse Cell Analyzer (Millipore), as previously described.^14^

### Cell invasion assay

Cell invasion was evaluated using Biocoat Matrigel invasion chambers and control inserts (Becton Dickinson Labware), as previously described.^14^ A total of 50,000 transfected cells/well were seeded in the invasion and control chambers, and 10% foetal bovine serum was used as the chemoattractant in the migration and invasion assays. Further information was described in Supplementary file.

### Migration scratch assay

MKN7 and NUGC cells (2 × 10^6^ cells/well) transfected with L1CAM siRNA or negative control siRNA in serum-free media were seeded into six-well plates and incubated for 12 h at 37 °C to attain confluence. Wounds were generated using a sterile 200-μl pipette tip, and wound closure was assessed using an Olympus IX71 microscope (Olympus, Center Valley, PA, USA) after 48 h incubation. Further information was described in Supplementary file.

### Anoikis assay

Anoikis assays were performed in six-well Costar Ultra-Low Attachment Microplates (Corning, NY, USA), as previously described.^17^ Further information was described in Supplementary file.

### Soft-agar colony formation assay

The base layer of soft agar contained complete DMEM media (10% FBS, 100 units per ml penicillin and 100 μg per ml streptomycin) with 0.6% agar; the top layer of soft agar contained complete DMEM media with 0.3% agarose and was mixed with 2500 cells per well of six-well plate and plated over the base layer, as previously described.^18^ The number of colonies was counted after 3 weeks.

### In vivo studies

Male nude mice (BALB/c) at 8 weeks of age were obtained from Japan SLC. The treatment protocol followed the guidelines for animal experimentation adopted by Mie University, and meets the standards required by the UKCCCR guidelines.^19^ To establish a mouse peritoneal metastasis model, NUGC3 gastric cancer cells (3 × 10^6^ cells/ml/mouse) transfected with L1CAM siRNA or negative control siRNA were injected intraperitoneally into mice under Isoflurane inhalation (Mylan, Tokyo, Japan), as previously described.^14^ Further information was described in Supplementary file.

### Statistical methods

Statistical analysis was performed using Medcalc version 16.8.4 (Mariakerke, Belgium). Differences between groups were estimated by Wilcoxon’s signed rank test, χ^2^ test, and one-way analysis of variance as appropriate. Overall survival (OS) was measured from the date of gastrectomy to the date of death from any cause, or last known follow-up for patients still alive. Disease-free survival (DFS) was measured from the date of surgery to the date of disease recurrence, death from any cause (i.e., non-cancer deaths were not censored) or until last contact with the patients. Receiver operating characteristic (ROC) curves were established for determining cut-off values for analysing each outcome (OS, DFS, lymph node metastasis, and distant metastasis) by Youden’s index. For time-to-event analyses, survival estimates were calculated with the Kaplan–Meier analysis, and groups were compared with the log-rank test. Cox’s proportional hazard regression test with stepwise regression was used to estimate univariate and multivariate hazard ratios for prognosis. Logistic regression analysis with stepwise regression was used to predict the factors influencing lymph node and distant metastasis. For multivariate testing, all clinicopathological parameters significant in univariate analysis were included. Clinical variables that were considered for univariate and multivariate analyses, in addition to target L1CAM expression status, were previously identified confounding factors that affected the prognosis and metastasis in patients with GC: sex, age at diagnosis, histological type (intestinal or diffuse), T stage (T1/2 or T3/4), venous invasion (present or absent), lymphatic vessel invasion (present or absent), nerve invasion (present or absent), lymph node metastasis (present or absent), and distant metastasis (presence or absence).

To clarify the prognostic risk of L1CAM expression in GC patients, we conducted propensity score matching (PSM) analysis. High or low expression of L1CAM protein in GC tissues was designated as the objective factor. Applying logistic regression analysis, a continuous propensity score ranging from 0 to 1 was generated. Matched covariates included age (<68 or ≥68 years), T classification (T1/2 or T3/4), venous invasion (presence or absence), lymphatic vessel invasion (presence or absence), nerve invasion (present or absent), lymph node metastasis (presence or absence), and distant metastasis (presence or absence), according to the results of the univariate analysis for risk of high L1CAM expression in GC tissues. Matching on the estimated propensity scores with null difference yielded 116 matched pairs with high or low L1CAM expression (58 patients in each group, *P* = 1.0). All *p* values were 2-sided, and values <0.05 were considered statistically significant.

## Results

### Expression of L1CAM protein in GC cells compared to cancer stroma or adjacent normal mucosa

**Fig. 1:**
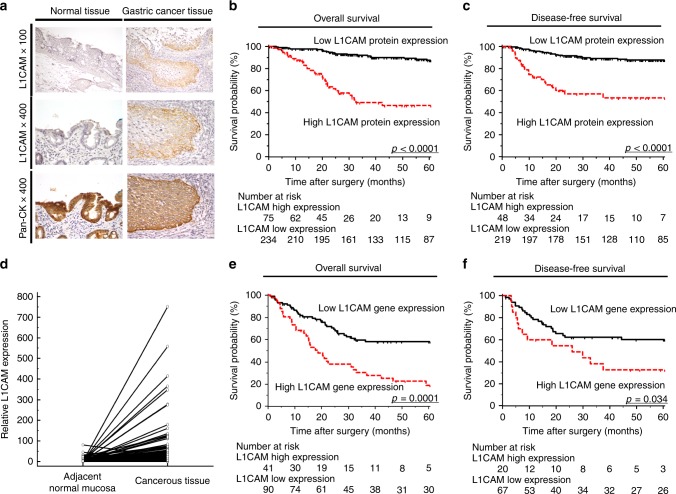
Prognostic impact of L1 cell adhesion molecule (L1CAM) expression status in overall survival (OS) and disease-free survival (DFS) of gastric cancer (GC) patients. **a** Immunohistochemical analysis of L1CAM expression in GC tissues and adjacent normal mucosa. **b, c** Kaplan–Meier survival curves for OS (**b**) and DFS (**c**) in GC patients based on expression of L1CAM in the formalin-fixed, paraffin-embedded cohort. OS and DFS in GC patients with high L1CAM expression in GC tissue were significantly lower than in those with low L1CAM expression (OS; *P* < 0.0001, DFS; *P* < 0.0001, log-rank test). **d** L1CAM expression was significantly elevated in GC tissues compared with adjacent normal mucosa in the fresh frozen cohort (*P* < 0.0001). **e**, **f** Kaplan–Meier survival curves for OS (**e**) and DFS (**f**) in GC patients based on L1CAM expression in the fresh frozen cohort. Expression status of L1CAM was significantly correlated with poor OS and DFS in fresh frozen cohort (OS; *P* = 0.0001, DFS; *P* = 0.034, log-rank test). All statistical tests were two-sided.

We performed immunohistochemical analysis for L1CAM and pan-cytokeratin using FFPE cohort specimens to investigate the cellular distribution of L1CAM protein expression in GC tissues. L1CAM protein expression was mainly observed in the membrane and occasionally in the cytoplasm of tumour cells. L1CAM protein expression was not observed in cancer stoma or normal gastric mucosa (Fig. 1a).

### L1CAM expression was associated with tumour malignancy and poor outcomes in the FFPE cohort of gastric cancer patients

We evaluated associations between protein expression and clinicopathological data in the FFPE cohort. According to the ROC analyses with Youden’s index correction for L1CAM expression analysis, we defined a cut-off value of >0 as the high-staining group (*n* = 75) and ≤0 as the low-staining group (*n* = 234). The high-staining group of L1CAM was significantly associated with old age (*P* = 0.001), advanced T stage (*P* < 0.0001), presence of venous invasion (*p* < 0.0001), lymphatic vessel invasion (*p* < 0.0001), nerve invasion (*p* < 0.0001), lymph node metastasis (*P* < 0.0001), and distant metastasis (*P* < 0.0001) in GC patients in the FFPE cohort (Table 1).

**Table 1:** Association between L1CAM protein expression and clinicopathological characteristics in FFPE cohort.

Variables	L1CAM expression		*P*
	High	Low	
	(*n* = 75)^a^	(*n* = 234)^a^	
Age			
≦68^b^	30	145	0.001*
>68^b^	45	89	
Gender			
Male	59	159	0.08
Female	16	75	
Histology			
Intestinal type	53	144	0.15
Diffuse type	22	90	
T classification			
T1/T2	19	151	<0.0001*
T3/T4	56	83	
Venous invasion			
Present	52	84	<0.0001*
Absent	23	150	
Lymphatic invasion			
Present	65	148	<0.0001*
Absent	10	86	
Nerve invasion			
Present	59	114	<0.0001*
Absent	14	113	
Lymph node metastasis			
Present	52	78	<0.0001*
Absent	23	156	
Distant metastasis			
Present	27	14	<0.0001*
Absent	48	220	

*L1CAM* L1 cell adhesion molecule, *FFPE* formalin-fixed and paraffin-embedded

*Bold values indicate *p* <   0.05

^a^Cut-off threshold of L1CAM was determined by receiver operating characteristic analysis with Youden’s index for overall survival in this cohort

^b^The median age at surgery was 69 years in this cohort

### High expression of L1CAM protein was an independent prognostic factor for OS and DFS in GC patients

We generated a Kaplan–Meier survival curve subdivided by L1CAM expression levels to perform time-to-event analysis and evaluated the potential use of L1CAM expression as a prognostic biomarker. High L1CAM expression was significantly correlated with poor prognosis, in terms of OS and DFS, compared with low expression (OS; *P* < 0.0001, Fig. 1b; DFS; *P* < 0.0001, Fig. 1c). Multivariate analysis revealed that elevated L1CAM expression was an independent prognostic factor for both OS (hazard ratio (HR), 2.42; 95% confidence interval (CI), 1.31–4.58; *p* = 0.005, Table 2a) and DFS (HR, 2.55; 95% CI, 1.33–4.87; *p* = 0.047, Table 2b).

**Table 2:** Multivariate analysis for predictors of (a) overall survival; (b) disease-free survival; (c) lymph node metastasis; (d) distant metastasis in FFPE cohort of gastric cancer patients.

Variables	Univariate			Multivariate		
	HR	95% CI	*P*	HR	95% CI	*P*
(a)						
Age (>68)	2.02	1.18–3.46	0.01*	1.35	0.77–2.36	0.3
Gender (male)	1.33	0.72–2.44	0.36			
Histology (diffuse)	0.9	0.52–1.58	0.72			
T classification (T3/4)	12.4	5.3–29.1	<0.0001*	1.69	0.51–5.63	0.39
Venous invasion (present)	6.53	3.29–13	<0.0001*	0.96	0.43–2.12	0.91
Lymphovascular invasion (present)	28.1	3.89–203	0.0009*	4.35	0.48–39.3	0.19
Nerve invasion (present)	14.6	4.54–46.7	<0.0001*	1.42	0.29–6.99	0.66
Lymph node metastasis (present)	14.8	6.32–34.6	<0.0001*	3.87	1.5–10	0.005*
Distant metastasis (present)	11.2	6.46–19.5	<0.0001*	3.24	1.78–5.9	0.0001*
L1CAM expression (high)	6.79	3.92–11.8	<0.0001*	2.45	1.31–4.58	0.005*

(b)						
Age (>68)	2.21	1.23–3.96	0.008*	1.24	0.66–2.34	0.51
Gender (male)	1.93	0.93–4.02	0.08			
Histology (diffuse)	1.03	0.56–1.88	0.93			
T classification (T3/4)	29.1	9.01–93.9	<0.0001*	9.23	1.73–49.2	0.009*
Venous invasion (present)	5.73	2.9–11.3	<0.0001*	1.03	0.46–2.3	0.94
Lymphovascular invasion (present)	13.7	3.32–56.6	0.0003*	1.67	0.33–8.5	0.33
Nerve invasion (present)	45.3	6.23–328	0.0002*	3.26	0.31–34.8	0.33
Lymph node metastasis (present)	13.1	5.87–29.5	<0.0001*	2.74	1.15–6.5	0.023*
L1CAM expression (high)	5.5	3.05–9.91	<0.0001*	2.55	1.33–4.87	0.047*

Variables	Univariate			Multivariate		
	OR	95% CI	*P*	OR	95% CI	*P*
(c)						
Age (>68)	1.51	0.96–2.38	0.08			
Gender (male)	2.45	1.44–4.17	0.001*	3.12	1.57–6.19	0.001*
Histology (diffuse)	0.74	0.46–1.19	0.22			
T classification (T3/4)	14	8.04–24.3	<0.0001*	3.87	1.76–8.49	0.0007*
Venous invasion (present)	8.52	5.08–14.3	<0.0001*	1.27	0.62–2.58	0.51
Lymphovascular invasion (present)	45.8	14–149	<0.0001*	16.5	4.38–62.4	<0.0001*
Nerve invasion (present)	14.8	7.84–28	<0.0001*	1.69	0.69–4.18	0.25
L1CAM expression (high)	4.52	2.58–7.92	<0.0001*	2.18	1.03–4.63	0.042*

(d)						
Age (>68)	1.44	0.75–2.78	0.28			
Gender (male)	1.16	0.55–2.43	0.69			
Histology (diffuse)	0.9	0.45–1.8	0.76			
T classification (T3/4)	32.8	7.74–138	<0.0001*	9.49	1.18–76.4	0.035*
Venous invasion (present)	12.1	4.6–31.8	<0.0001*	3.07	1.02–9.21	0.046*
Lymphovascular invasion (present)	–	–	0.99			
Nerve invasion (present)	18.2	4.3–76.9	0.0001*	0.69	0.08–5.93	0.73
Lymph node metastasis (present)	13.3	5.6–35.1	<0.0001*	3.1	1.02–9.37	0.045*
L1CAM expression (high)	8.84	4.32–18.1	<0.0001*	3.88	1.74–8.65	0.0009*

The median age at surgery is 68 years.

Cut-off threshold of L1CAM was determined by ROC analysis with Youden's index for OS in (a); DFS in (b); lymph node metastasis in (c); and distant metastasis in (d)

*HR* Hazard ratio, *OR* Odds ratio

*Bold values indicate *p* < 0.05

### High L1CAM expression in GC tissues is a predictive factor for lymph node and distant metastasis in the FFPE cohort

We performed multivariate logistic analysis to assess predictive potential of L1CAM expression for lymph node and distant metastasis in GC patients. Interestingly, elevated L1CAM expression in GC tissues was an independent risk factor for both lymph node metastasis (odds ratio (OR), 2.18; 95% CI, 1.03–4.63; *p* = 0.0042, Table 2c) and distant metastasis (OR, 3.88; 95% CI, 1.74–8.65; *p* = 0.0009, Table 2d). Collectively, these data highlight that assessment of L1CAM protein expression in GC tissues might be used as a predictive biomarker for distant metastasis in GC patients.

### Prognostic impact of L1CAM expression was successfully validated using PSM analysis in GC patients

Recent evidence has demonstrated PSM analysis as a new statistical method for overcoming different patients’ characteristics and selection bias to increase the evidence level of a nonrandomised observational study.^20^ To validate the prognostic potential of L1CAM expression in GC patients, we conducted PSM analysis using FFPE cohort, and yielded 116 GC patients (58 patients in each group) for further analysis. Inter-group differences were not found for any of the clinicopathological factors. Survival curve analysis showed that GC patients with high L1CAM expression demonstrated poorer OS (*P* = 0.009, Supplementary Fig. 1a) and DFS (*P* = 0.013, Supplementary Fig. 1b) compared with patients with low L1CAM expression.

### Clinical impact of L1CAM gene expression using fresh frozen specimens was consistent with the findings from the FFPE cohort

We successfully identified and validated the biomarker potential of L1CAM expression for identification of high-risk population for oncological outcomes in GC patients. However, quantification of L1CAM gene expression using preoperative biopsy specimens might be better suited in the clinical setting. If successful, this would provide physicians with valuable information to decide upon the treatment course for GC. Considering that such biopsy specimens are generally preserved in a fresh frozen state, we next investigated whether L1CAM expression in fresh frozen specimens could be used to identify high-risk subsets for various oncological outcomes, including lymph node or distant metastasis, recurrence, and survival in GC patients before surgery. Expression levels of L1CAM in 131 GC tissues and paired adjacent normal mucosal tissues were examined by quantitative RT-PCR. Expression of L1CAM was significantly elevated in GC tissues compared with matching adjacent normal mucosa (*P* < 0.0001; Fig. 1d). We analysed the expression patterns of L1CAM with various clinicopathological factors to determine whether L1CAM expression has any prognostic significance in GC patients (Supplementary Table 1). The expression cut-off thresholds for L1CAM were determined according to ROC analyses with Youden’s index to determine OS of GC patients. Tumours with high L1CAM expression had more lymph node metastasis (*P* = 0.003) and distant metastasis (*P* = 0.007) than those with low L1CAM expression. These results demonstrated that the clinical impact of L1CAM gene expression in the fresh frozen cohort was consistent with that of protein expression in the FFPE cohort.

To validate the predictive potential of L1CAM expression based on data from FFPE cohort for determining GC prognosis, we evaluated whether gene expression of L1CAM in fresh frozen specimens could predict prognosis in GC patients. Patients with elevated L1CAM expression in GC tissues had significantly poorer prognosis than those with decreased L1CAM expression below the cut-off point in terms of OS and DFS in fresh frozen cohort (OS: *p* = 0.0001, Fig. 1e; DFS: *p* = 0.034, Fig. 1f). Multivariate Cox regression analysis revealed that elevated L1CAM expression was an independent prognostic factor for OS (HR, 1.96; 95% CI, 1.19–3.23; *p* = 0.008, Table 3a), and DFS (HR, 2.26; 95% CI, 1.02–5.01; *p* = 0.044, Table 3b) in fresh frozen cohort.

**Table 3:** Multivariate analysis for predictors of (a) overall survival; (b) disease-free survival; (c) lymph node metastasis; (d) distant metastasis in a fresh frozen cohort of gastric cancer patients.

Variables	Univariate			Multivariate		
	HR	95% CI	*P*	HR	95% CI	*P*
(a)						
Age (>69)	1.6	0.98–2.61	0.06			
Gender (male)	0.58	0.34–1.01	0.06			
Histology (diffuse)	1.31	0.8–2.2	0.31			
T classification (T3/4)	3.28	1.75–6.15	0.0002*	2.28	1.2–4.34	0.012*
Venous invasion (present)	4.27	1.55–11.7	0.005*	2.04	0.71–5.81	0.18
Lymphovascular invasion (present)	3.41	0.83–13.9	0.09			
Nerve invasion (present)	1.39	0.6–3.27	0.44			
Lymph node metastasis (present)	5.7	2.29–14.2	0.0002*	3.89	1.54–9.82	0.004*
Distant metastasis (present)	4.34	2.64–7.14	<0.0001*	2.86	1.69–4.83	0.0001*
L1CAM expression (high)	2.63	1.62–4.26	0.0001*	1.96	1.19–3.23	0.008*

(b)						
Age (>69)	1.15	0.61–2.18	0.66			
Gender (male)	0.97	0.41–2.33	0.95			
Histology (diffuse)	1.61	0.82–3.14	0.17			
T classification (T3/4)	4.03	1.84–8.83	0.0005*	3.54	1.58–7.89	0.002*
Venous invasion (present)	2.08	0.87–4.98	0.1			
Lymphovascular invasion (present)	2.76	0.66–11.5	0.16			
Nerve invasion (present)	1.73	0.61–4.92	0.3			
Lymph node metastasis (present)	5.29	1.87–14.9	0.002*	3.38	1.17–9.77	0.025*
L1CAM expression (high)	2.29	1.05–5.01	0.037*	2.26	1.02–5.01	0.044*

Variables	Univariate			Multivariate		
	OR	95% CI	*P*	OR	95% CI	*P*
(c)						
Age (>69)	1.69	0.75–3.82	0.21			
Gender (male)	1.39	0.54–3.57	0.49			
Histology (diffuse)	0.56	0.23–1.27	0.17			
T classification (T3/4)	3.28	1.43–7.56	0.005*	1.86	0.63–5.5	0.26
Venous invasion (present)	5.68	2.15–15.1	0.005*	2.57	0.68–9.8	0.17
Lymphovascular invasion (present)	9.43	2.27–39.2	0.002*	4.6	0.72–29.2	0.11
Nerve invasion (present)	3.37	1.11–10.3	0.03*	2.05	0.54–7.87	0.29
L1CAM expression (high)	6.22	1.77–21.9	0.004*	4.11	1.03–16.4	0.045*

(d)						
Age (>69)	1.53	0.71–3.31	0.28			
Gender (male)	0.29	0.12–0.69	0.005*	0.22	0.08–0.62	0.004*
Histology (diffuse)	2.23	0.99–5.02	0.05			
T classification (T3/4)	4.54	1.62–12.7	0.004*	4.09	1.35–12.3	0.013*
Venous invasion (present)	–	–	0.99			
Lymphovascular invasion (present)	3.81	0.47–31.2	0.21			
Nerve invasion (present)	2.88	0.61–13.5	0.18			
Lymph node metastasis (present)	3.32	1.07–10.3	0.037*	2.53	0.67–9.5	0.17
L1CAM expression (high)	5.6	1.83–17.1	0.003*	5.47	1.66–18.1	0.005*

The median age at surgery is 69 years.

Cut-off threshold of L1CAM were determined by ROC analysis with Youden's index for OS in (a); DFS in (b); Lymph node metastasis in (c); Distant metastasis in (d)

*HR* Hazard ratio, *OR* Odds ratio

*Bold values indicate *p* < 0.05

### High L1CAM expression in GC tissues is a predictive factor for lymph node and distant metastasis in the fresh frozen cohort

To understand further the predictive potential of L1CAM expression using fresh frozen specimens from GC patients, we performed multivariate logistic analysis to examine the predictive value of L1CAM expression in GC tissues for lymph node and distant metastasis (Table 3c and d). Surprisingly, elevated L1CAM expression in GC tissues was an independent predictive factor for lymph node metastasis (OR, 4.11; 95% CI, 1.03–16.4; *p* = 0.045, Table 3c) and distant metastasis (OR, 5.47; 95% CI, 1.66–18.1; *p* = 0.005, Table 3d) in GC tissues. Collectively, these results suggest that L1CAM expression in fresh frozen specimens is a clinically feasible biomarker to identify patients at high risk of recurrence and poor prognosis and support close correlation between L1CAM expression and GC progression.

### L1CAM expression in GC cell lines

**Fig. 2:**
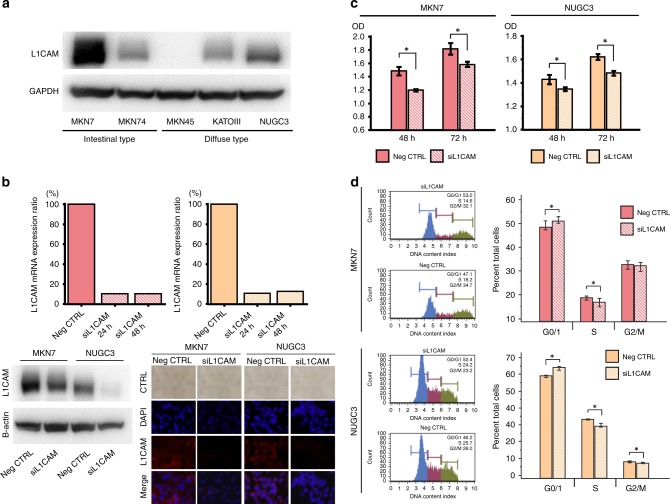
Series of in vitro analyses in gastric cancer (GC) cell lines treated with L1 cell adhesion molecule (L1CAM)**. a** Western blotting analysis to detect L1CAM transcript expression in GC cell lines. **b** L1CAM expression knockdown in MKN7 and NUGC3 cells. GC cells were transfected with either L1CAM-siRNA (siL1CAM) or negative control siRNA (Neg CTRL). The mRNA expression ratio of siL1CAM to Neg CTRL was measured 24 and 48 h after transfection by quantitative RT-PCR (Upper). Western blotting analysis demonstrated that L1CAM protein expression was also suppressed in L1CAM siRNA-treated GC cells compared to those with negative control siRNA transfection (Left Lower). Immunofluorescence analysis also verified the results of qPCR or western blotting analysis (Right Lower). **c** Assay after 48 and 72 h to investigate the proliferative effects of L1CAM in GC cells. The proliferation rate was significantly impaired after L1CAM knockdown compared to Neg CTRL cells. **d** Cell cycle analysis demonstrated that the G0/G1-phase fraction was significantly increased after L1CAM knockdown in both cell lines.

As described above, overexpression of L1CAM was correlated with progression and metastasis of GC, as well as high risk of recurrence and survival. In view of these findings, we determined the functional role of L1CAM in the pathogenesis of GC. We investigated L1CAM expression by western blotting analysis in established GC cell lines (Fig. 2a). MKN7 and NUGC3 cells showed highest L1CAM expression. In all other cell lines, L1CAM expression was markedly lower. Based on these results, we selected MKN7 and NUGC3 cell lines for further knockdown experiments. Transfection of GC cell lines with L1CAM siRNA resulted in significant reduction in L1CAM mRNA expression (up to 90%) compared with negative control siRNA-treated cells 24 h post-transfection (Fig. 2b). Furthermore, both of western blotting analysis and Immunofluorescence analysis clearly verified the qPCR results and showed that L1CAM protein expression was decreased in both GC cells with L1CAM siRNA transfection compared to those with negative control siRNA transfection (Fig. 2b).

### L1CAM promotes proliferation, cycle progress, invasion and migration in GC cells

**Fig. 3:**
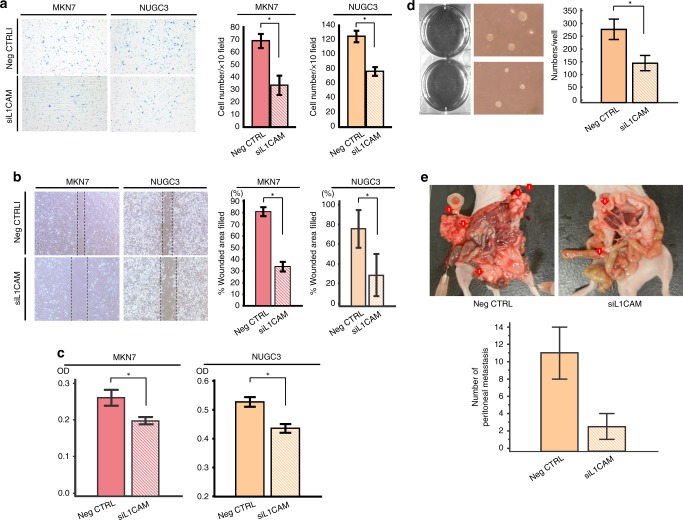
Series of in vitro and in vivo analyses in gastric cancer (GC) cell lines treated with L1 cell adhesion molecule (L1CAM). **a** Invasion assays demonstrated that the capacity of L1CAM-siRNA-transfected GC cells to invade through a Matrigel-coated membrane was significantly reduced compared to that of Neg-CTRL-transfected cells. **b** Migration scratch assays treated with or without L1CAM knockdown. Knockdown of L1CAM expression significantly reduced cancer cell migration in both GC cells. **c** Assay to investigate the anoikis resistance of GC cells after treatment with or without L1CAM knockdown. After anoikis induction for 48 h, an assay was performed and the number of viable floating cancer cells in low-attachment plates was calculated by MTT assay. **d** Soft-agar colony formation assay demonstrated that there was a significant decrease in the number of colonies of NUGC3 cells with siL1CAM transfection when compared to NUGC3 cells.

We assessed various cellular functions such as proliferation and invasion after treatment with non-silencing siRNA and L1CAM siRNA. Downregulation of L1CAM resulted in significant inhibition of tumour cell growth at 48 and 72 h after L1CAM siRNA transfection (Fig. 2c). Cell cycle analysis demonstrated that the G0/G1-phase fraction was significantly increased after L1CAM knockdown in both cell lines (Fig. 2d). We next performed invasion and migration scratch assays to determine whether attenuated L1CAM levels affected cellular invasion and migration. L1CAM siRNA transfection of MKN7 and NUGC3 GC cells showed reduced invasive and migration capacity compared with cells transfected with non-silencing siRNA (Fig. 3a, b). Taken together, these results demonstrate that L1CAM expression induces concurrent proliferation, invasion, and migration in GC cells.

### L1CAM enhances anoikis resistance and colony formation in an anchorage-independent manner

Anoikis is currently recognised as an apoptotic process by loss of cell adhesion,^21^ and therefore, anoikis resistance is involved in the metastatic process.^22^ Our clinical findings revealed that L1CAM overexpression was an independent risk factor for distant metastasis; therefore, we hypothesised that L1CAM has the function of resistance to anoikis in GC cells. To elucidate whether inhibition of L1CAM induced anoikis, we evaluated by MTT assay the number of viable MKN7 and NUGC3 cells that were floating in low-attachment plates. L1CAM knockdown caused a decrease in the number of viable GC cells, which was significantly lower than the number of viable cells transfected with non-silencing siRNA (Fig. 3c). Furthermore, to assess whether inhibition of L1CAM could suppress colony formation in an anchorage-independent manner, we conducted soft-agar colony formation assay. In these culture conditions, there was a significant decrease in the number of colonies of NUGC3 cells with siL1CAM transfection when compared to NUGC3 cells with negative control transfection (Fig. 3d).

### Inhibition of L1CAM expression suppresses formation of peritoneal metastasis mouse models

To assess whether knockdown of L1CAM suppresses the formation of peritoneal dissemination in GC, we administered NUGC3 cells transfected with either L1CAM siRNA or negative control siRNA intraperitoneally into nude mice (3 × 10^6^ cells per mouse). Intriguingly, numbers of peritoneal tumours following L1CAM siRNA transfection were drastically fewer than in mice transfected with control siRNA (Fig. 3e).

## Discussion

Development of lymph node or distant metastasis has a major effect on recurrence and prognosis in patients with cancer. Identification of patients at high risk for lymph node and distant metastasis could help oncologists with treatment decision-making and improve prognosis of GC. Recent evidence has demonstrated L1CAM as an oncogenic driver in various malignancies,^23–27^ and the clinical burden and prognostic biomarker potential of L1CAM expression evaluated by immunohistochemistry has been shown in several cancers, including GC. However, to date, no study has shown that L1CAM can be used for preoperative prediction of risk for recurrence, lymph node metastasis and distant metastasis.

In this study, we systemically investigated the potential role of L1CAM in GC development, and made several novel discoveries. First, L1CAM protein was mainly expressed in tumour cells of GC tissues, and high L1CAM expression was an independent risk factor for both recurrence and survival in the FFPE cohort. Second, L1CAM expression in GC tissues was an independent risk factor for lymph node and distant metastasis in GC patients. Third, PSM analysis revealed the predictive potential of L1CAM expression for unfavourable oncological outcomes in GC patients. Fourth, we validated the prognostic impact and predictive potential of L1CAM expression for distant metastasis using fresh frozen specimens from GC patients. Fifth, inhibition of L1CAM expression suppressed invasion and migration capacity of GC cells, as well as proliferation, cycle progress, anoikis resistance and tumorigenicity in an anchorage-independent manner. Finally, knockdown of L1CAM inhibited the formation of metastatic nodules in a peritoneal metastasis model.

Despite recent advances in diagnostic techniques and chemotherapy in GC, one-third of patients will have reached an advanced stage with distant metastasis at the time of diagnosis, and prognosis remains poor.^28,29^ Furthermore, lymph node metastasis is also recognised as a critical risk factor for recurrence in GC patients receiving curative surgery.^30^ Therefore, elucidation of pivotal factors involved in the metastatic process might be used as new prognostic markers for early detection of recurrence and improvement of prognosis in GC patients. L1CAM is one of the transmembrane adhesion molecules, and several lines of evidence show that L1CAM overexpression is significantly correlated with metastasis-related clinicopathological factors and unfavourable outcome in various malignancies. Tischler and co-workers evaluated L1CAM protein expression in 468 patients with non-small cell lung cancer, and demonstrated that high L1CAM expression was significantly correlated with distant metastasis, and an independent prognostic factor for OS.^31^ Consistent with these findings, one of the major results in our study was the clinical impact of L1CAM expression in GC patients. Elevated expression of L1CAM was significantly correlated with lymph node and distant metastasis in the FFPE cohort. Furthermore, increased L1CAM expression was an independent prognostic factor for both DFS and OS. Collectively, our data suggest that L1CAM is involved in the metastatic process in GC progression, and could be a feasible predictor of oncological outcome.

Another major finding of this study is biological role of L1CAM expression in GC development. In addition to the findings from multivariate analysis, we conducted PSM to clarify the prognostic impact of L1CAM expression in relation to the different background characteristics of the patients. This novel statistical method clearly demonstrated that GC patients with L1CAM overexpression showed poorer DFS and OS compared to those with low L1CAM expression, regardless of TNM staging. These findings suggest the potential oncogenic role of L1CAM at any time during GC development. Therefore, we also performed functional analyses of L1CAM in GC cells in vitro and in vivo. Several evidences have demonstrated L1CAM as an oncogenic driver in various types of cancer.^32–34^ Hai and colleagues used short-hairpin RNA to show that inhibition of L1CAM expression significantly suppressed invasiveness and tumorigenicity of lung cancer cells via the extracellular signal-regulated kinase pathway.^32^ Consistent with these findings, we revealed that L1CAM knockdown inhibited various oncogenic phenotypes related to the metastatic process, including proliferation, cycle progress, invasion, migration, and anoikis resistance in GC cell lines. These findings clearly suggest that L1CAM affects the proliferation and invasiveness of the primary GC and sustains the antiapoptotic state of detached cells as they disseminate to metastatic sites and promote viable lymph node or distant metastases.

In the clinical setting, one of the major issues for molecular tests is the availability of appropriate specimens for evaluation. In particular, the different characteristics of FFPE and fresh frozen specimens cannot be ignored in clinical tests. The development of quantitative techniques using fresh frozen specimens could create a path toward preoperative tests in GC patients who need treatment decision-making. With regard to clinical tests, our study validated the predictive value of L1CAM expression for metastasis and poor oncological outcomes using fresh frozen specimens from GC patients. Regional lymph node metastasis mainly affects the prognosis of GC patients undergoing curative resection. Recent advances in endoscopic techniques, including endoscopic mucosal or submucosal dissection, could provide curative treatment for GC patients without lymph node metastasis. In contrast, distant metastasis remains a life-threatening event in GC patients, and availability of predictive biomarkers for distant metastasis is critical in the overall management of advanced GC. In this scenario, our novel findings suggest that assessment of L1CAM expression using fresh frozen specimens might support physicians and patients to decide upon treatment strategy preoperatively.

## Conclusion

Our findings show that L1CAM expression may serve as an important biomarker for identification of GC patients who are at high risk for poor oncological outcomes, and as a therapeutic target. Furthermore, preoperative evaluation of L1CAM gene expression using biopsy samples could help to decide upon neoadjuvant treatment in the future.

## Supplementary information

Supplementary file

## Data Availability

Primary research data are presented in a summative fashion in the manuscript. No publicly available datasets have been generated as part of this work.
