# Supplementary material for: Clinical significance and biological role of L1 cell adhesion molecule in gastric cancer
Source: Br J Cancer. 2019 Nov 22;121(12):1058–68. doi: 10.1038/s41416-019-0646-8 (PMC6964673; doi:10.1038/s41416-019-0646-8)

**Supplementary File**

**Clinical significance and biological role of L1 cell adhesion molecule in gastric cancer**

**Running tit**le: L1CAM predicts gastric cancer survival

Takashi Ichikawa M.D.^1*^, Yoshinaga Okugawa M.D. Ph.D.^1*^, Yuji Toiyama M.D. Ph.D.^1^, Koji Tanaka M.D. Ph.D.^1^, Chengzeng Yin M.D.^1^, Takahito Kitajima M.D.Ph.D.^1^, Satoru Kondo M.D. Ph.D.^1^, Tadanobu Shimura M.D. Ph.D.^1^, Masaki Ohi M.D. Ph.D.^1^, Toshimitsu Araki M.D. Ph.D.^1^,Masato Kusunoki M.D. Ph.D.^1^

^1^Department of Gastrointestinal and Pediatric Surgery, Mie University Graduate School of Medicine, Tsu, Japan

***** T. Ichikawa, and Y. Okugawa contributed equally to this work.

**Supplementary Material and Methods**

*Cell proliferation assay*

Cell proliferation was evaluated using a WST-8 [2-(2-methoxy-4-nitrophenyl)-3-(4-nitrophenyl)-5-(2, 4-disulfophenyl)-2H-tetrazolium, monosodium salt] colorimetric assay. Twenty-four hours after reverse transfection, L1CAM siRNA-transfected and negative-control-siRNA-transfected cells (5000 cells/well) were seeded onto 96-well cell plates (Becton Dickinson Labware, Franklin Lakes, NJ) in 100 μl culture medium. After 0–72-h culture, the medium was discarded and replaced with 90 μl fresh medium, followed by addition of 10 μl WST-8 reagent solution (Cell Counting Kit; Dojindo Laboratories, Kumamoto, Japan) and incubated for 2 h at 37°C. Each independent experiment was performed three times. Cell proliferation was determined by colorimetric comparison by reading OD values from a microplate reader (SoftMax; Molecular Devices, San Jose, CA, USA) at an absorption wavelength of 450 nm.

*Cell invasion assay*

Cell invasion was evaluated using Biocoat Matrigel invasion chambers and control inserts (Becton Dickinson Labware), as previously described^14^. A total of 50 000 transfected cells/well were seeded in the invasion and control chambers, and 10% fetal bovine serum was used as the chemoattractant in the migration and invasion assays. The Matrigel invasion chambers and control inserts were incubated for 24 h at 37°C. The incubation medium containing cells was removed from the top chamber using cotton swabs and serum-free medium. The membranes were fixed in methanol, stained with Mayer’s hematoxylin, dehydrated in ethanol, and mounted on glass slides. The number of cells that invaded the underside of the membrane was then determined. Each independent experiment was performed three times.

*Migration scratch assay*

MKN7 and NUGC cells (2×10^6^ cells/well) transfected with L1CAM siRNA or negative control siRNA in serum-free media were seeded into 6-well plates and incubated for 12 h at 37°C to attain confluence. Wounds were generated using a sterile 200-μl pipette tip. The cells were then incubated at 37°C for an additional 48 h. Wound closure was assessed using an Olympus IX71 microscope (Olympus, Center Valley, PA, USA) at 10× magnification. Cell migration distance was measured using Adobe Photoshop 9.0.2 software and compared with baseline measurements. Each independent experiment was performed at least three times.

*Anoikis assay*

Anoikis assays were performed in six-well Costar Ultra-Low Attachment Microplates (Corning, NY, USA), as previously described^15^. L1CAM siRNA-transfected or negative-control-siRNA-transfected cells were suspended in RPMI-1640 with L1CAM siRNA or negative control siRNA at a concentration of 5×10^5^ cells/ml. Cell suspensions (2 ml) were added to each well and incubated for 24 h in a humidified atmosphere (37°C and 5% CO_2_). After induction of anoikis, MTT assay was performed with cells seeded at 5 × 10^3^ cells/well in microtiter plates (96 wells, flat bottom) in a final volume of 100 µl culture medium per well. Spectrophotometric absorbance of the samples was measured as described above. Each independent experiment was performed three times.

*In vivo studies*

Male nude mice (BALB/c) at 8 weeks of age were obtained from Japan SLC. Mice have free access to water and food, housed in pathogen-free cages containing wood shavings and bedding in a 12-h light/dark cycle, with controlled room temperature (RT). The treatment protocol followed the guidelines for animal experimentation adopted by Mie University, and meets the standards required by the UKCCCR guidelines^17^. To establish a mouse peritoneal metastasis model, NUGC3 gastric cancer cells (3×10^6^ cells/ml/mouse) transfected with L1CAM siRNA or negative control siRNA were injected intraperitoneally into mice under Isoflurane inhalation (Mylan, Tokyo, Japan), as previously described^14^. Two mice were used in each group. To examine the effect of L1CAM inhibition on the peritoneal dissemination potential of gastric cancer cells, mice were euthanized using Isoflurane inhalation in their home cages, followed by cervical dislocation to ensure death on six weeks tumour cell postinjection. We evaluated the number of nodules in the mesentery and peritoneal walls of each mouse.

**Supplementary Figure 1**

**Potential of L1 cell adhesion molecule (L1CAM) expression as a prognostic marker using propensity score matching (PSM) analysis. (a)** Survival curve analysis subdivided by L1CAM expression in gastric cancer tissues using PSM analysis demonstrated that elevated L1CAM expression was significantly correlated with poor overall survival (P=0.009, log-rank test, **(a)** and disease-free survival (P=0.013, log-rank test, **(b)**. All statistical tests were two-sided. *p <0.05.

| **Supplementary Table 1** |  |  |  |
| --- | --- | --- | --- |
| **Association between L1CAM gene expression and clinicopathological characteristics in fresh frozen cohort** | | | |
|  |  |  |  |
| Variables | L1CAM expression | | P |
|  | High | Low |  |
|  | (n=41) | (n=90) |  |
| Age |  |  |  |
| ≦69^#^ | 17 | 46 | 0.31 |
| ＞69^#^ | 24 | 44 |  |
| Gender |  |  | 0.57 |
| Male | 31 | 72 |  |
| Female | 10 | 18 |  |
| Histology |  |  | 0.07 |
| Intestinal type | 25 | 69 |  |
| Diffuse type | 16 | 21 |  |
| T classification |  |  | 0.27 |
| T1/T2 | 11 | 33 |  |
| T3/T4 | 30 | 57 |  |
| Venous invasion |  |  | 0.15 |
| Present | 37 | 72 |  |
| Absent | 4 | 18 |  |
| Lymphatic invasion |  |  | 0.13 |
| Present | 40 | 81 |  |
| Absent | 1 | 9 |  |
| Nerve invasion |  |  | 0.31 |
| Present | 30 | 66 |  |
| Absent | 3 | 13 |  |
| Lymph node metastasis |  |  | **0.003*** |
| Present | 38 | 62 |  |
| Absent | 3 | 28 |  |
| Distant metastasis |  |  | **0.007*** |
| Present | 18 | 19 |  |
| Absent | 23 | 71 |  |
| ^#^The median age at surgery is 69 years in this cohort. | | | *** p<0.05** |
| ^†^Cut-off threshold of L1CAM was determined by receiver operating characteristic analysis with Youden's index for overall survival in this cohort.  Abbreviations: L1CAM, L1 cell adhesion molecule. | | | |
|  |  |  |  |


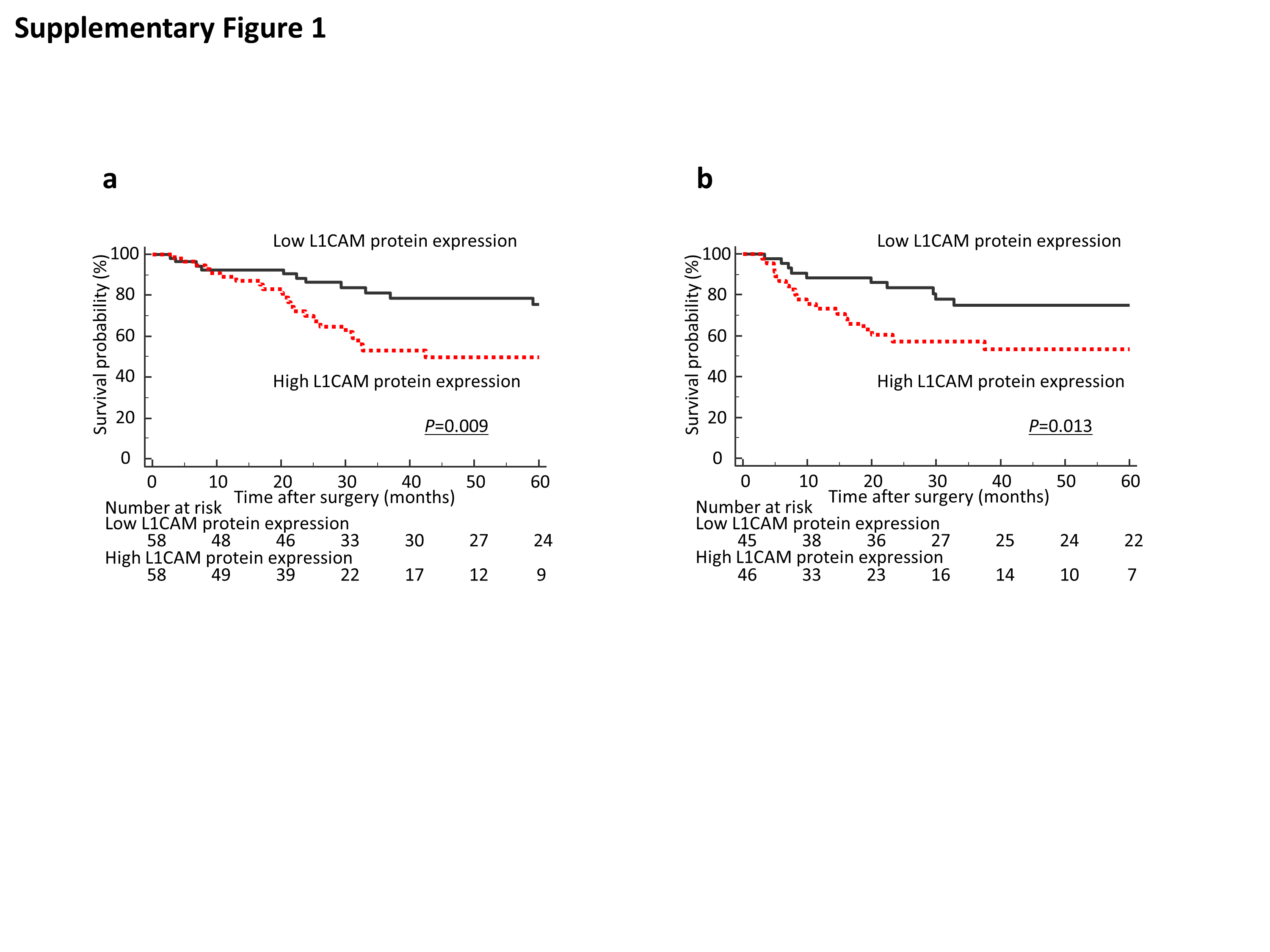

Supplement: Supplementary file 1 — Supplementary file [file 41416_2019_646_MOESM1_ESM.docx]
